# An Approach to Automated Fusion System Design and Adaptation

**DOI:** 10.3390/s17030601

**Published:** 2017-03-16

**Authors:** Alexander Fritze, Uwe Mönks, Christoph-Alexander Holst, Volker Lohweg

**Affiliations:** InIT—Institute Industrial IT, Ostwestfalen-Lippe University of Applied Sciences, 32657 Lemgo, Germany; alexander.fritze@hs-owl.de (A.F.); christoph-alexander.holst@hs-owl.de (C.-A.H.); volker.lohweg@hs-owl.de (V.L.)

**Keywords:** information fusion, sensor fusion, intelligent sensor, self-configuration, knowledge-based system

## Abstract

Industrial applications are in transition towards modular and flexible architectures that are capable of self-configuration and -optimisation. This is due to the demand of mass customisation and the increasing complexity of industrial systems. The conversion to modular systems is related to challenges in all disciplines. Consequently, diverse tasks such as information processing, extensive networking, or system monitoring using sensor and information fusion systems need to be reconsidered. The focus of this contribution is on distributed sensor and information fusion systems for system monitoring, which must reflect the increasing flexibility of fusion systems. This contribution thus proposes an approach, which relies on a network of self-descriptive intelligent sensor nodes, for the automatic design and update of sensor and information fusion systems. This article encompasses the fusion system configuration and adaptation as well as communication aspects. Manual interaction with the flexibly changing system is reduced to a minimum.

## 1. Introduction

Sensors and actuators, but also other sources such as databases serve as data sources for the realisation of condition monitoring in industrial applications or for the acquisition of characteristic parameters, such as production speed or rejection rate. The data originates from sources which are spatially distributed over the shop floor. Modern industrial plants are equipped with an increasing number of sensors generating a large amount of data. The task of processing these large amounts becomes increasingly complex. Computation takes longer and necessary communication may exceed the available bandwidth. Furthermore, machine operators are unable to properly process and draw correct conclusions from the generated information [1]. The issues caused by the increased complexity are addressed by *Sensor and Information Fusion* (SEFU/IFU) mechanisms. These collect and combine data and information from different sources to reduce complexity as well as uncertainty. The resulting fused information is of higher value and precision than the information gained by the individual single sources. The idea of SEFU/IFU is to create new or more precise knowledge about the system’s environment or status (such as physical quantities or occurring events) by taking into consideration different information sources [2].

As of today, the design, update, and adaptation of SEFU/IFU processes remain major open topics. The design of robust monitoring systems requires a system designer to fully and properly understand the functionalities of the monitored system. Complex machineries make it increasingly difficult for a system designer to comprehend the overall industrial plant. In such complex systems, the acquisition of sensor signals must be designed very carefully and tailored optimally towards the specific application. This challenge is aggravated by the progressing introduction of modular and flexible systems and devices. In current industrial plants, the idea of flexible systems and devices is realised only partly, especially at runtime. Flexibility is often only pre-designed, which demands a designer to consider all possible situations beforehand. However, this poses a huge challenge on the system designer in addition to that of the increasing complexity of the monitored systems. Hence, automatic fusion system design methods are sought-after. In the current state-of-the-art, no methodologies, frameworks, or tool-chains for designing and restructuring information processing and fusion systems are available, either open or free, although conceptual techniques are published [3,4,5]. These works propose completely automated concepts which are not considered optimal, since SEFU/IFU system designers may feel disregarded in the decision process of designing fusion systems. Instead, a design methodology is suggested in this article. It automatically designs a SEFU/IFU system, but gives the system designer the final authority over the actual implementation. The methodology relies on a rule-based decision system, which evaluates semantic descriptions delivered by the involved sensors. This work briefly covers the concept of Multi-Agent Systems. These are covered in the state-of-the-art and their advantages and limitations compared to the proposed approach are given. In addition, the proposed SEFU/IFU system design is implemented and evaluated in a condition monitoring application. The condition monitoring is carried out with the *Multilayer Attribute-based Conflict-reducing Observation System* (MACRO) information fusion system [6].

This article is based on the previous conference contribution [7]. It is extended by recent research with respect to real-world implementations. Aspects considering auto-configuration by the application of agent-based methods are further included. The article is structured as follows. After the introductory section, related work in the scope of SEFU/IFU (including the MACRO system) and orchestration approaches is presented in Section 2. Section 3 presents the proposed SEFU/IFU design approach, whose implementation is described in Section 4. Evaluation results in the scope of the aforementioned condition monitoring application under laboratory conditions are presented in Section 5 before the article is concluded in Section 6.

## 2. Related Work

Before related work in the context of information fusion systems design is presented, the general constraints of SEFU/IFU are summarised in the following.

Hall and Llinas describe that the aim of SEFU/IFU is to optimise “the accuracy of applications” [2] (p. 1). Which criterion is to be optimised needs to be defined before the SEFU/IFU application is designed. Only then can meaningful signal sources, which are able to obtain data containing the required information to derive a precise and accurate statement about the criterion, be chosen. As described in [8], if two sensors $S_{1}$ and $S_{2}$ work with complementary physical principles, the combined *performance* $\mathrm{Perf}(S_{1}\cup S_{2})$ regarding the chosen criterion will be increased, such that

$$\mathrm{Perf}\left(S_{1}\cup S_{2}\right)>\mathrm{Perf}\left(S_{1}\right)+\mathrm{Perf}\left(S_{2}\right),\;\mathrm{or}\;\mathrm{at}\;\mathrm{least}\text{:}\;\;\mathrm{Perf}\left(S_{1}\cup S_{2}\right)>\max\left(\mathrm{Perf}\left(S_{1}\right),\mathrm{Perf}\left(S_{2}\right)\right).$$

Furthermore, SEFU/IFU approaches must consider the following constraints, which apply to typical application scenarios of systems in the field of machine and plant engineering [6]:

**Data source heterogeneity:** The sources delivering the data, which describe the current situation, are of many kinds. These include, among others:

- technical sensor units (e.g., temperature, pressure, humidity sensors),
- multimodal sensor units (e.g., audio-visual camera systems),
- database systems (storing, e.g., past measurements, production plans),
- expert knowledge.

**Data type heterogeneity:** The data is of dimension n∈N where each source may have its own dimensionality. The types of data representing measurement quantities are manifold. Occurrences of their characteristics are listed in Table 1, of which every arbitrary combination is possible.

**Data uncertainty:** The acquired data is prone to uncertainties, which are categorised to aleatory (noise, random variations, material characteristics, etc.) and epistemic (incompleteness, imprecision, production tolerances, etc.) uncertainty according to the following definitions [9]:

**Definition 1** (Aleatory uncertainty)**.** Aleatory uncertainty is characterised by its random and non-deterministic nature and thus represents the inherent randomness of a problem.

**Definition 2** (Epistemic uncertainty)**.** *Epistemic uncertainty is also denoted by* subjective uncertainty*. Its source is the lack of knowledge due to, e.g., incomplete data.*

Effects resulting from uncertainties can also lead to conflicts in the acquired data.

**Data volume:** The amount of acquired data increases continuously due to an increasing number of sources in the systems. Reasons include

- availability of new sources through sub-system inclusion,
- utilisation of actuators as sensors (such as shared use of data for motion control and condition monitoring),
- exploitation of new data source concepts (e.g., utilisation of consumer-market smartphones for vibration measurements [10]).

**Spatial data source distribution:** Large industrial applications require the distribution of their sub-systems over the shop floor. The data sources available in these sub-systems are consequently also spatially distributed. In order to obtain a complete overview of the entire system, all data needs to be collected and aggregated.

One information fusion system taking the above constraints into account is the MACRO system. It is applied as an exemplary SEFU/IFU approach in this article and is introduced in the next subsection.

### 2.1. Multilayer Attribute-Based Conflict-Reducing Observation (MACRO)

**Figure 1:**
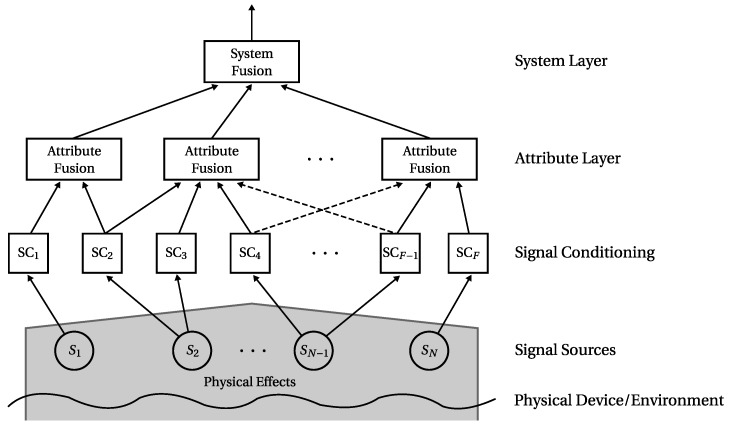
Multilayer attribute-based conflict-reducing observation system MACRO [16].

The *Multilayer Attribute-based Conflict-reducing Observation System* (MACRO) information fusion approach is applied for the fusion of several sensor signal inputs. Its basic concept is summarised in the following. The entire theoretical background is elaborated in [6,11]. MACRO’s structure is depicted in Figure 1.

The MACRO fusion structure consists of multiple layers. This structure is inspired by the decision-making process of social groups of humans: Individuals (humans/sensors) discuss their opinions (measurements) in groups (attribute layer). This process is susceptible to conflicts inside the groups. MACRO is specifically designed to consider and reduce conflicts in the information fusion process. The resulting information of the group discussion is, again similar to decision-making in social groups, combined at organisational level (system layer) to derive a global decision. For more information on the human group decision-making background of MACRO, the reader is referred to [6,12]. It was shown that the application of this approach for condition monitoring purposes is beneficial compared to state-of-the-art approaches [6,11,13].

The MACRO fusion approach for the determination of a system’s global state is carried out as follows: In a first step, signals from the system as well as from its environment (such as temperature, electric current or pressure) are acquired by sensors, i.e., *signal sources*. In the following *signal conditioning* step, features are extracted from the signals. The signal conditioning may also include signal preprocessing procedures. Multiple features may be extracted from one single signal, e.g., features which determine the mean and variance of a signal (cf. Figure 1). Without loss of generality, the following assumes one feature per signal. Sensor measurements obtained in the signal conditioning step may include all sorts of (physically) different types of quantities (e.g., temperature, air pressure). These measurements are transformed into a unitless space. A *fuzzy set theory* [14] approach based on [15] has been chosen for modelling the acquired data in a common unitless space between 0 and 1 [11]. MACRO then combines the conditioned signals into groups denoted by *attributes* at the attribute layer. Attributes are defined as follows:

**Definition** **3** (Attribute [7])**.** *An attribute represents a characteristic (physical quantity, functionality, component, etc.) of the monitored system that is represented by at least two features. The attributes depend both on the monitored system and the application in which MACRO is utilised, and are defined by experts’ knowledge. Given the hierarchy of the monitored system, four types of attributes are defined in the following taxonomy:*

**Module attribute:** An attribute is a module attribute if it represents a single module or component that is part of the monitored system.

**Physical attribute:** An attribute is a physical attribute if it characterises a single elementary (physical, biological, chemical) phenomenon of a specific module.

**Functional attribute:** An attribute is a functional attribute if it characterises functionality of the monitored entity with respect to a specific module.

**Quality attribute:** An attribute is a quality attribute if it assesses the output (e.g., fabricated product) of the monitored system.

An attribute’s output indicates to which degree its inputs represent the system’s normal condition and is denoted by attribute health.

Thus, each attribute is related to the monitored physical system by its semantic meaning. The attributes are manually defined in the fusion system design process depending on the specific application. At least two information sources are combined to one attribute. This redundancy is exploited for both (i) detecting sensor faults and defects as well as (ii) cross-checking the consistency of sensor values.

The subsequent fusion of the attributes’ health is carried out at the system layer. It determines the system health in order to examine the entire supervised system.

Detailed information regarding MACRO and its constituent parts is found in [6,11,12,17]. This contribution concentrates on the automatic orchestration of SEFU/IFU systems, based on available signal sources and feature extraction algorithms. MACRO is utilised as an example fusion system in this scope for validation purposes.

### 2.2. System Design and Configuration

Concepts that deal with the challenge of self-organisation and tasks such as feature identification are utilised in various works. An approach for adaptive condition monitoring of heterogeneous components is described in [18]. The authors apply MACRO fusion and extend it by automated attribute generation and update functionality according to the current system structure. Another concept of Chakraborty and Pal [19] (extended in [20]) utilises *artificial neural networks* in the form of a *modified radial basis function network* as well as a *multilayered perceptron network* [20] for selecting useful groups of features. The selection and orchestration of information sources is carried out manually. Subsequently, an optimisation is applied to identify and reject improper information sources. Iswandy and König have proposed a framework and methodology for the design of intelligent multi-sensor systems [3]. The system design is split into local tasks such as sensor parameterisation, signal conditioning, feature selection, etc. For each task, a sequential optimisation is carried out based on evolutionary algorithms, in particular *genetic algorithms* and *particle swarm optimisation*. An exemplary application for this concept is an *intelligent spoon* for *smart-kitchens* or medical use cases [5,21].

Another approach for automated fusion system generation is the application of a *middleware*. It interconnects processes by abstraction [22]. Processes are capable of operating at different levels. A middleware, which focuses on the discovery and selection of sensors, is presented by Alex et al. [23]. The authors model the sensor selection process based on *Bayesian* and *decision theoretical* paradigms [24]. Another approach incorporating *context* for system composition is presented in [22]. Context denotes the circumstance or situation of the task (location, temperature, etc.). For some specific context, the approach identifies suitable information sources. Aspects such as self-configuration, -healing, and -optimisation are considered as well. *Nexus* is a middleware that consists of an *expressive description framework* to semantically annotate services [25]. It aims at structure discovery and information fusion. A general middleware, which acts as server-client architecture and which is not restricted to any specific application domain, is the *Open Platform Communication Unified Architecture* (OPC UA). OPC UA is platform-independent and capable of cross-networking. It implements a generic information model, which determines how to access information as well as its inherent structure (representation) [26].

Other research, which facilitates automated SEFU/IFU system design, is carried out in the field of *semantic technologies.* Semantic annotations enable to automatically discover, invoke, compose, and monitor entities by applying knowledge inference [27]. Semantic technologies mainly arose from the research area of *Semantic Web Services* (SWS). To ensure interoperability, SWSs extend web services to include machine-interpretable semantics for reasoning. A general overview about the capabilities of SWSs in factory automation is presented by Lastra and Delamer [27] . Knowledge has to be modelled appropriately first to apply semantic concepts and to describe the underlying concept by predefined objects and relations between them. A well-known approach to this is the concept of *ontologies*. They include definitions of a shared vocabulary (syntax) and relations between specific terms/symbols (semantics) [28]. An overview of present ontologies targeting semantic specifications of sensors is given in [29]. Compton et al. present an approach for sensor composition, which relies on the *Web Ontology Language* (OWL), i.e., a common knowledge representation language [30]. Further modelling languages exist that allow to describe sensors or other entities in a predefined manner.

The *Automation Markup Language* (AutomationML), for example, is a description language that enables storage of manufacturing system process data following an object-oriented approach. It includes engineering information such as system topologies, geometries, kinematics, etc. [31]. The *Sensor Model Language* (SensorML) is a common language for the description of sensors and their internal processes [32]. SensorML is an *eXtensible Markup Language* (XML)-based language mainly applied for semantic web-based applications, but is also applicable to other areas. It allows to describe solitary sensors, but also multi-sensor systems. Furthermore, non-physical functionalities such as filters can be modelled. In addition, SensorML is capable of modelling complex process chains and algorithms [32,33]. Semantics are also applied for sensor networks (i.e., a collection of sensor nodes that consist of a processor unit, communication modules, and power supply) to facilitate automated sensor network configuration and resource-constrained planning (addressing energy consumption, lifetime, coverage). An approach to self-configuration and role assignment in sensor networks with respect to consistent and resource-efficient communication is presented by Frank and Römer [34]. Rybicki and Domaszewicz also focus on sensor networks, but try to identify available composite services (sensors) [35]. Loskyll et al. show more specific concepts for semantic-based service discovery and orchestration in industrial applications [36]. For service discovery, the system reads a user-specified *semantic template* and checks it against a predefined ontology. Bröring et al. introduce a semantic plug-and-play concept for the Sensor Web [33]. They propose a complete process chain covering the range from heterogeneous sensors up to the application layer. Sensors are described by a common description language in combination with external ontologies. The authors make use of and extend the concept of *Sensor Bus* that acts as middleware. This middleware only focuses on sensor discovery and data exchange and does not consider orchestration aspects.

As of today, it is increasingly common that sensors are equipped with communication capabilities and local processing power [16]. The available processing capacities enable the incorporation of local intelligence, which in turn is necessary for a collective of devices to self-organise themselves. A *self-organising system* is a system that operates without any central control unit or global state repository [37,38]. Such a system is completely decentralised. Any decision made by the system, either on a course of action or a change of internal organisation, is the result of collective cooperation. Self-organising systems possess several advantages over systems with a centralised intelligence. A central decision unit always presents a single point of failure and a system without such a unit is overall less error-prone and more robust [39]. Self-organising systems scale better in comparison and adapt themselves better to changing environments. Consequently, they are more suitable for ad hoc networks and networks which change often in their structure and number of participants. The trade-off for these advantages are: (i) self-organising systems tend to produce more communication load for the organisation process [37]; (ii) designing such systems requires an increased effort [40]; and (iii) depending on applied strategies, they are less deterministic [37].

A promising tool to build a self-organising distributed system is the concept of *agents*. A definition of an agent is given by Wooldridge:

**Definition** **4** (Agent [41])**.** *An agent is a computer system that is situated in some environment, and that is capable of autonomous action in this environment in order to meet its design objectives.*

To achieve its design objectives, an agent relies on autonomous behaviour, social ability, reactivity, and pro-activeness [42]. Autonomous behaviour means that an agent takes decisions and performs actions without external control. Social ability comprises more than just communicating with other entities. An agent may not (and probably will not) reach its design goal alone with its own resources caused by lack of information, knowledge, sensors or actuators [43]. Hence, it needs to cooperate with others, asking them to provide missing resources and negotiate with them. The term “reactivity” refers to the ability to respond to changes in an agent’s environment or internal states. Finally, pro-activeness describes that an agent does not solely react, but rather tries to fulfil its goals by taking pre-emptive measures. Agents are categorised in two types depending on whether an agent is exclusively software-based or if it has a physical body through which it is able to interact with its physical environment. Consequently, software-based agents are referred to as *software agents* [44]. An agent situated in a physical environment consists of a physical body with sensors and actuators to interact with this environment. These kinds of agents are examples of an embodiment as described in [45]. In swarm robotic science, they are called *embodied agents* [46].

A survey of self-organisation mechanisms which are developed for use in a *Multi-Agent System* (MAS) is presented in [47]. A MAS is a collective of agents, which combine their efforts to work towards an overall goal [48]. For this, they communicate, negotiate, and cooperate with each other. The capabilities of a MAS exceed those of each individual agent. Bajo et al. propose a concept in which fusion of sensor data is organised using software agents [49]. The authors focus on an agent-based implementation of information fusion techniques. They assume that data from a sensor network is already gathered on a central device, but do not consider a self-organisation of distributed sensor nodes. Frei and Serugendo utilise embodied agents to organise an assembly system in [50]. The authors focus on the self-organisation of the assembly system, but do not explicitly incorporate information fusion methods. An overview of state-of-the-art industrial applications of MASs is given in [51]. Leitão et al. point out that MAS technologies gain increasing acceptance in industrial applications. They are mostly utilised for high-level tasks, such as scheduling and planning of the industrial process, machine monitoring, and self-organisation. Currently, MASs are only rarely used for direct process control or for implementations demanding hard real-time constraints.

Altogether, different approaches exist that try to tackle the problem of the automated design of adaptive SEFU/IFU systems. However, none of the concepts mentioned here serves as a tool to support the design process with respect to industrial applications. Furthermore, the configuration of proper real-time communication channels for process data exchange in connection with an automatic design procedure is not considered in related works. Therefore, this contribution proposes a support system that focuses on these aspects.

## 3. Automated Fusion System Design

In this section, a concept to overcome the problem of complex and error-prone SEFU/IFU system design is introduced that relies on previous work of [7,52]. Sensors have to be capable of information processing as well as exchange of configuration data for the self-configuration of SEFU/IFU systems. Following these requirements, *intelligent sensors* are incorporated for this work. An intelligent sensor is defined as follows:

**Definition** **5** (Intelligent Sensor [6,7])**.** *An intelligent sensor is a modular component with the following characteristics:*

- It is equipped with one or more elementary sensors, memory, and one or more processor units, as well as communication interfaces.
- An intelligent sensor is self-adaptable, i.e., its parameters (measurement range, accuracy, etc.) change with respect to changes in the environment.
- *The functionalities of an intelligent sensor are distributed over the following layers:*
  – The application layer implements signal processing capabilities containing, among others, feature extraction on the basis of raw sensor data as well as SEFU/IFU implementations to generate high-level information.
  – The middleware layer abstracts the connectivity layer from the application layer, and includes a self-description that relies on a defined data structure and vocabulary from a shared knowledge base.
  – The connectivity layer implements the communication interfaces and fulfils the requirements for intelligent networking (auto-configuration, adaptability, etc.).

Intelligent sensors are able to improve the situation regarding the dilemma of a complex, time-consuming, and error-prone system design and are therefore an essential part of the developed methodology.

With respect to the human system designer and operator, a SEFU/IFU system has to be transparent, understandable, and traceable. These properties allow erroneous situations to be properly detected and resolved. Consequently, the following requirements for a design methodology are derived [6]:

- The application’s requirements have to be fulfilled by a proper selection of sensors with respect to the measured quantity, the measurement range, and resolution. A general intelligent sensor according to Definition 5 is suggested that adapts to the actual condition and automatically operates in the optimal configuration. Furthermore, the intelligent sensor includes a self-description for automated fusion system design.
- The system designer should only be assisted in the design process and must remain as the final decision maker. Solutions for the design should, at most, be suggested such that the system designer can choose the most appropriate one.
- Each design of a SEFU/IFU system depends on the specific application. Nevertheless, partial solutions are reusable and should therefore be considered before identifying a completely new SEFU/IFU system design. Consequently, repositories for storage of problem formulations and solutions have to be available that hold information in a defined manner to identify similarities.
- Attributes of the MACRO system or their input signals include descriptive information to automatically generate, update, and destroy the attributes. Hence, available autoconfiguration mechanisms have to be extended by a fusion system design methodology in order to be able to process self-descriptive data that originates from intelligent sensors.

This work especially considers the possible orchestration and configuration of a SEFU/IFU system. In the following, the architecture of the automated design methodology is introduced. Thereupon, the theoretical background and the general orchestration procedure are discussed.

### 3.1. System Architecture

**Figure 2:**
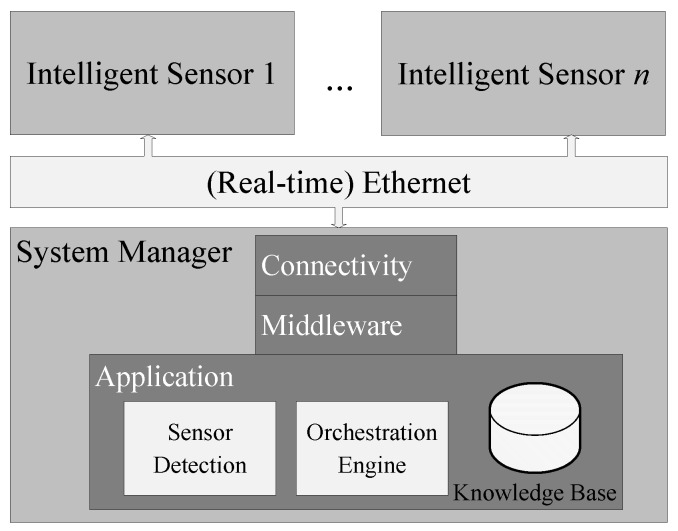
Architecture of the fusion system [52].

The architecture of the SEFU/IFU system is depicted in Figure 2. It forms a sensor network consisting of *n* intelligent sensors that are capable of communicating among themselves and with the *system manager*. The system manager implements functionalities for automated system design and self-configuration.

In this case, the system manager is a central processing unit. In Section 2, distributed systems in the form of MASs and their advantages regarding self-organisation and adaptation are introduced. Because of better scaling and the avoidance of a single point of failure, such systems fit also into the concept of automated fusion system design. Nonetheless, industrial applications require real-time communication channels for process data exchange to be able to react to changes in process real-time. Thus, it must also be considered in SEFU/IFU. There is currently no real-time communication standard that is capable of fulfilling the requirements of decentralised data exchange. Therefore, a central processing unit is indispensable when real-time communication is required. Consequently, the concept presented in the following additionally incorporates a central processing unit in the form of the system manager.

Furthermore, the system manager discovers changes in the fusion system architecture. This part of the system manager is referred to as *sensor detection*. Intelligent sensors may be attached to or detached from the system. For example, a new intelligent sensor which is plugged to the network is detected automatically and its semantic self-description is read. The description contains information about available elementary sensors or available signal processing algorithms and is automatically transferred to the *Knowledge Base* (KB) [52]. The detection procedure is further detailed in Section 4. The KB consists of domain-specific information (such as available intelligent sensors, their equipment, a shared vocabulary, the hierarchy of the monitored system). The orchestration engine utilises information of the KB to orchestrate the SEFU/IFU system. Relations between information sources and available signal processing algorithms are inferred to define the fusion system configuration. The orchestration engine is carried out as a *knowledge-based system*. Knowledge-based systems have gained importance since the early 1960s and serve as a tool to represent and process human knowledge on machines. The two main aspects of knowledge-based systems are *knowledge representation* and *knowledge processing* [53]. To make use of available knowledge, it has to be modelled with respect to a defined form of representation. Only then do knowledge processing techniques allow for inference of new knowledge.

In the presented case, the knowledge-based system is implemented as a *rule-based system*. It is introduced in the following section before the actual orchestration of the SEFU/IFU is discussed.

### 3.2. Rule-Based Systems

Rule-based systems are well-established concepts for both knowledge modelling and inference. They were first proposed by Post [54]. In his work, he focused on a computing model for production systems, which in this case characterise a rule-based system. Such systems consist of *productions*, i.e., predefined rules. In this case, rules represent available knowledge in the form of conditional sentences, as they are also used in natural human language. The structure of such rules is as follows [53,55]:

ifXthenY.

Here, *X* and *Y* are two valued logic expressions that represent the *condition* and the conclusion. The latter follows if *X* is fulfilled. Formally, *X* represents an *object* and its *value*, which are connected by some *operator* (e.g., x≤5.) [55]. Therefore, the condition is a logic expression that can be modelled by formal logics such as *propositional logic* or *first-order logic* [56].

This work makes use of propositional logic and relies on *atomic sentences* (e.g., $A,\;P,\;Warm,\;X_{a}$) that represent single propositions. Sentences are mapped to the set $\left\{0,1\right\}$ where 0 characterises the logic proposition false, while 1 states that the proposition is true. To assign a meaning (piece of knowledge) to an atomic sentence *X*, the following notion is used:

X:=“It is dark”.

A combination of multiple atomic sentences by some operators is referred to as *complex sentence*. Available operators are the negation (¬), conjunction (∧), disjunction (∨), implication (→), and equivalence (↔) operators [24]. This propositional logic operator syntax is used in the following to define sentences that represent knowledge of a certain domain. Consider two atomic sentences *X* and *Y*. Following the previously introduced notation, a rule (*r*) can be formulated as follows:

r:X→Y.

This rule is also referred to as *simple rule* because it consists of a single conclusion only [53,56]. *Complex rules* consist of complex sentences in their premise and conclusion (e.g., $r:X_{1}\wedge X_{2}\vee X_{3}\to Y_{1}\vee Y_{2}$). This representation is unfavourable as it aggravates the subsequent inference process. For efficient knowledge derivation, only simple rules are suitable. Rules have either to be defined based on this restriction or, if they are complex rules, have to be transformed into simple rules. A transformation technique is available from [53]. To formulate available knowledge about the modelled process, a set of rules (R) is defined. Inference is drawn by a combination of facts $b_{i},\;i=\left\{1,\ldots ,n\right\}$ that form the *fact base* $B=\{b_{1},\ldots ,b_{n}\}$ and the rule base R. Facts provide actual information about the modelled process and are used to check whether rules are fulfilled or not, i.e., rules imply new facts by given facts. Hence, inference is drawn by deduction. The underlying method for inference is the *modus ponens* [24]. The modus ponens is written as follows:

$$\begin{array}{cc} r:X\to Y & \\ X=\mathrm{true} & \left(\mathrm{fact}\right)\\ Y=\mathrm{true} & (\mathrm{inference}). \end{array}$$

Having a set of rules, techniques are required to iterate over the complete rule base. For the purpose of this work, *forward chaining* is applied. The rule base R and the fact base B are initially given. Using forward chaining, the modus ponens is sequentially applied to every rule and the fact base B is extended by the conclusion if inferred. At this point, the necessity for a transformation of complex rules into a set of simple rules becomes obvious. If the conclusion consists of a complex sentence instead of single literals, B cannot be extended offhand. The algorithm iterates over the rule base R as long as B is extended by new conclusions. The process of forward chaining is depicted in Algorithm 1 [24,53].

**Algorithm 1:** Forward Chaining Algorithm
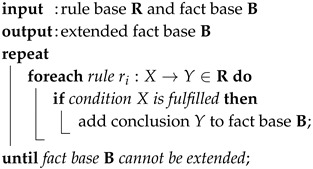


The orchestration procedure for the automated design of the SEFU/IFU system is carried out following the concept of rule-based systems. This is discussed in the following section.

### 3.3. Orchestration

The orchestration process aims to provide a possible solution for the design of a SEFU/IFU system. With respect to the MACRO fusion system, this includes the following tasks:

1. Initialisation of attributes.
2. Inference of features.
3. Assignment of features to attributes.

First, attributes are either deduced automatically or specified by means of experts’ knowledge. For feature inference, combinations of available solid sensors $S_{j},\;j=\left\{1,\cdots ,m\right\}$ and algorithms $A_{k},\;k=\left\{1,\cdots ,l\right\}$ are evaluated. Available features are finally mapped to available attributes resulting in a proper SEFU/IFU system. These tasks are detailed in the following sections.

#### 3.3.1. Attribute Initialisation

Attributes are denoted by $a_{i},\;i=\left\{1,\cdots ,n\right\}$ and are pooled in an *attribute set* A. They underlie a predefined taxonomy of attributes according to Definition 3. Two strategies are applied for the initialisation of attributes. First, module, physical, and quality attributes are deduced with respect to the system set-up and the set of available sensors. Second, the system designer has the possibility to manually initialise functional attributes. Attribute information that is modelled and stored in the KB is:

- A *Unique Identifier* (UID),
- The attribute type (physical, module, quality, functional),
- An associated object,
- A set of physical phenomena $p=\{p_{1},\ldots ,p_{s}\}$,
- A set of allowed features $f=\{f_{1},\ldots ,f_{t}\}$.

The UID is a unique text string for attribute identification. The attribute type indicates the particular classification of an attribute. The associated object represents the entity that an attribute characterises. This either is a specific module or the output (product) of the monitored entity. Therefore, the hierarchy of the observed system has to be available from the KB and is modelled by experts’ knowledge. For example, the associated object can represent complete system modules such as a printing station, a single component such as a motor, or the output of the printing unit. The elements of the sets p and f are text strings (terms), which are taken from the defined vocabulary. These sets are required for the later orchestration procedure and state the allowed physical phenomena and features for the respective attributes.

Module and physical attributes as well as one quality attribute are deduced automatically. Every module of the observed entity is necessary for the functionality of the overall system. Hence, the orchestration engine runs through the system hierarchy and initialises one attribute for each module. Therefore, the hierarchy has to be modelled before the actual orchestration is carried out. Modules are observed by heterogeneous sensors that measure versatile physical phenomena. Therefore, physical attributes, which state a specific module, are also generated automatically. The sensors’ self-descriptions include the information about the associated object. Thus, in connection with the system hierarchy, the KB entails information about available physical characteristics for each module. The initialisation procedure of module and physical attributes is depicted in Algorithm 2.

**Algorithm 2:** Initialisation of Module and Physical Attributes
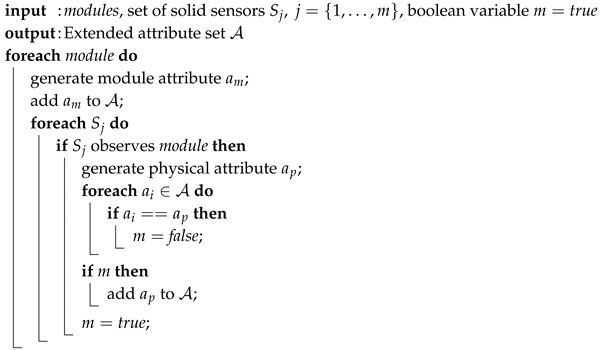


For module attributes, the sets of suitable physical phenomena p and features f are empty sets (p=∅, f=∅). There is no restriction regarding the later attribute assignment and every feature is suited to be assigned to the attribute as long as it states the specific module (cf. Section 3.3.3).

For physical attributes, the cardinality of set p is |p|=1. Hence, p consists only of a single entry: the physical phenomenon that a sensor measures. The set of suitable features is empty (f=∅) as it is not required for the subsequent orchestration (cf. Section 3.3.3).

**Example** **1.** *Consider a press-module with the UID “*inIT:modules:press*”. It forms metal and is part of a production system. Furthermore, consider that the press is observed by a temperature sensor. In this case, two attributes are automatically generated. These are the following:*

*1.*  *A module attribute that represents the module itself (*inIT:attributes:press*). This attribute consists of empty sets p=∅ and f=∅.*
*2.*  *A physical attribute for temperature. This attribute includes the module as an associated object (*inIT:modules:press*), the set of physical phenomena p={temperature}, and an empty set of suitable features f=∅.*

Furthermore, a quality attribute is automatically generated in the same way as module attributes are created. Its only observed entity is the product and it has no particular physical specification. Thus, again p=∅ and f=∅. Further quality attributes can be defined by experts’ knowledge.

Functional attributes are defined by experts’ knowledge as well. The observed system may show specific characteristics that are not deducible from available information of the KB. These characteristics have to be available as an attribute in order to obtain a robust and meaningful information fusion system. Therefore, the system operator can incorporate experts’ knowledge by formulating functional attributes.

**Example** **2.** *Consider the press-module described in Example 1. The press is equipped with a number of motors. By experts’ knowledge, the system designer models a functional attribute that represents the* running smoothness *of the press module. The set of allowed physical phenomena is p={temperature,currentconsumption,solid-bornesound,acoustic} and allowed features are f={mean,variance}.*

Attributes take feature values as the input. Therefore, possible features have to be identified first. The feature assignment is detailed in the following section.

#### 3.3.2. Feature Assignment

Intelligent sensors are equipped with solid sensors and algorithms, whereas features are combinations of both. For identification of matching sensors and algorithms, rules are defined and used as a tool for inference. Required information is available from the self-description of intelligent sensors and is therefore also available from the KB. Algorithms require specific input signals with a defined dimensionality. Restrictions may also apply, e.g., specific physical phenomena for which they are applicable. In addition, sensors provide similar information about their outputs. These are matched in order to deduce possible features for the overall fusion system.

With respect to the available solid sensors $S_{j},\;j=\left\{1,\ldots ,m\right\}$ and algorithms $A_{k},\;k=\left\{1,\ldots ,l\right\}$, the following propositions are defined:

(1) $$\begin{array}{cc} X_{A1}:= & “\text{The dimension of the output of }S_{i}\text{ and the input of }A_{k}\text{ are similar}”, \end{array}$$

(2) $$\begin{array}{cc} X_{A2}:= & “S_{j}\text{ observes a suitable physical phenomenon for }A_{k}”, \end{array}$$

(3) $$\begin{array}{cc} Y_{A}:= & “S_{j}\text{ matches to }A_{k}”. \end{array}$$

Based on the propositions of Equations (1)–(3) ,the general rule for feature inference is defined as follows:

$$r_{A}:X_{A1}\wedge X_{A2}\to Y_{A}.$$

The rule is constructed for all possible combinations of available algorithms $A_{k}$ and sensors $S_{j}$. If the sensor’s output and the algorithm’s input match and the physical phenomenon of the sensor fits to the algorithm, proposition $Y_{A}$ is inferred ($Y_{A}=\mathrm{true}$). Therefore, a feature $F_{v},\;v=\left\{1,\ldots ,w\right\}$ is added to the feature set F. Characteristics of a feature (e.g., its output dimensionality or the physical characteristic that it states) are derived from the sensor’s and algorithm’s characteristics.

The final task of the orchestration procedure is the attribute assignment that maps features to available attributes. This is detailed in the following section.

#### 3.3.3. Attribute Assignment

For the attribute assignment, similar rules as for the feature assignment are defined. Attributes have certain characteristics that have to be considered for rule definition (cf. Section 3.3.1). Therefore, rules are specifically defined for each attribute-type. Facts, which are required to prove whether rules are fulfilled, are derived from the description of available features and attributes.

Physical attributes state the condition of a specific observed module regarding a physical phenomenon. Therefore, to assign a feature to a physical attribute, both have to state the identical physical phenomenon and have to be associated to the same object. As mentioned before, the hierarchy of the monitored system is known. Furthermore, sub-components (e.g., a motor as a sub-component of a car) are related to the associated object. Consequently, features that state some sub-component must also be considered. Hence, the following facts are defined for inference of physical attributes:

$$\begin{array}{cc} X_{P1}:= & “a_{i}\text{ is of type physical}”,\\ X_{P2}:= & “F_{v}\text{ and }a_{i}\text{ have an equal associated object}”,\\ X_{P3}:= & “\text{The associated object of }F_{v}\text{ is a sub-component of the associated object of }a_{i}”,\\ X_{P4}:= & “F_{v}\text{ states about the physical phenomenon of }a_{i}”,\\ Y_{P}:= & “F_{v}\text{ fits to }a_{i}”. \end{array}$$

Using these facts, the defined rule for the assignment of a feature $F_{v}$ to some attribute $a_{i}$ is

$$r_{P}:X_{P1}\wedge \left(X_{P2}\vee X_{P3}\right)\wedge X_{P4}\to Y_{P}.$$

In order to assign features to functional attributes, different conditions have to be fulfilled. First, features have to state the same physical phenomenon as the attribute. Second, it is required that features are explicitly allowed for this attribute. This means that a feature $F_{v}$ has to be available in the set of allowed features f of an attribute $a_{i}$. These conditions are user-dependent because functional attributes are modelled by experts’ knowledge (cf. Section 3.3.1). Defined propositions for the assignment of features to functional attributes are the following:

$$\begin{array}{cc} X_{F1}:= & “a_{i}\text{ is of type functional}”,\\ X_{F2}:= & “F_{v}\text{ and }a_{i}\text{ have an equal associated object}”,\\ X_{F3}:= & “\text{The associated object of }F_{v}\text{ is a sub-component of the associated object of }a_{i}”,\\ X_{F4}:= & “F_{v}\text{ states about the physical phenomenon }a_{i}”,\\ X_{F5}:= & “F_{v}\text{ is allowed for }a_{i}”,\\ Y_{F}:= & “F_{v}\text{ fits to }a_{i}”. \end{array}$$

The general rule, which is available for all combinations of $F_{v}$ and $a_{i}$, is defined as

$$r_{F1}:X_{F1}\wedge \left(X_{F2}\vee X_{F3}\right)\wedge X_{F4}\wedge X_{F5}\to Y_{F}.$$

Less complex facts and rules are defined for the assignment of features to a module or to the quality attribute. In both cases, only the observed object should fit to the attribute’s associated object. For module attributes, this is a specific module and for the quality attribute it is the output of the monitored entity. For module attributes, required facts are

$$\begin{array}{cc} X_{M1}:= & “a_{i}\text{ is of type module}”,\\ X_{M2}:= & “F_{v}\text{ and }a_{i}\text{ have an equal associated object}”,\\ X_{M3}:= & “\text{The associated object of }F_{v}\text{ is a sub-component of the associated object of }a_{i}”,\\ Y_{M}:= & “F_{v}\text{ fits to }a_{i}”. \end{array}$$

The resulting rule is

$$r_{M}:X_{M1}\wedge \left(X_{M2}\vee X_{M3}\right)\to Y_{M}.$$

Facts defined for the quality attribute are

$$\begin{array}{cc} X_{Q1}:= & “a_{i}\text{ is of type quality}”,\\ X_{Q2}:= & “S_{j}\text{ and }a_{i}\text{ have an equal associated object}”,\\ Y_{Q}:= & “S_{j}\text{ fits to }a_{i}”. \end{array}$$

Therefore, the general rule for the assignment of sensors to a quality attribute is

$$r_{Q}:X_{Q1}\wedge X_{Q2}\to Y_{Q}.$$

Based on the aforementioned facts and rules, inference is drawn by the forward chaining algorithm (cf. Section 3.2). If some conclusion is inferred, the feature is assigned to the corresponding attribute. A set of associated features $C_{a_{i}}$, which holds associated features, is available after the algorithm has terminated. In order to apply SEFU/IFU, at least two input features for an attribute are required. Therefore, all attributes whose set of associated features is of cardinality $|C_{a_{i}}|\leq 1$ are rejected. Furthermore, it is possible that more than one attribute incorporate the same features. For example, if a motor is a sub-component of a cylinder and the motor is equipped with two temperature sensors, physical attributes for both objects are inferred. Attributes are compared to each other to avoid this case. If they incorporate one or more identical features, only the attribute that is associated to the lowest object in the system hierarchy is retained. Furthermore, functional attributes dominate physical attributes and physical attributes dominate module attributes.

The output of the orchestration engine is a set of attributes and their associated features. Therefore, the design of the SEFU/IFU system is complete with respect to its general structure. The result is proposed to the user as a possible design for the SEFU/IFU system. Besides the orchestration, the communication of (i) semantic information for knowledge inference and (ii) process data for the actual fusion is of interest. Therefore, the subsequent section details the actual implementation of the developed concept including those functionalities.

## 4. Implementation

Self-descriptive intelligent sensors and the orchestration procedure are the basic parts of the developed concept. Intelligent sensors according to Definition 5 are not yet available on the market. Hence, a prototypical implementation is carried out that relies on the *Raspberry Pi* as the evaluation platform [57]. The platform allows to attach more than one elementary sensor via several interfaces (SPI, USB, UART, etc.). Furthermore, functionalities for all functional layers can be implemented because local processing and memory units are available. For communication, an Ethernet interface is implemented. Details about the Raspberry Pi and involved software packages for the implementation are available from Appendix A. The prototypical implementation has been published to Zenodo (denoted by “Automated Fusion System Design and Adaptation Implementation”) for free access (doi:10.5281/zenodo.345130) [58].

The orchestration procedure connects available information automatically in order to derive a possible SEFU/IFU system. Additional functionalities are implemented for further improvements regarding adaptivity and communication. Besides the orchestration, information has to be modelled and communicated between available intelligent sensors and the system manager. Therefore, a middleware for device management and exchange of semantic information is incorporated. Furthermore, interfaces for real-time communication of process data are available from intelligent sensors and the system manager. The implementation of these components is outlined in the following sections.

### 4.1. Middleware

The middleware of an intelligent sensor is essential for the orchestration task. It holds semantic information of devices and manages the exchange of those information. OPC UA is applied here [59]. Its information model contains a network of nodes and defines the structure of modelled information. Nodes represent different entities that are physical or abstract. OPC UA servers include specific information models and OPC UA clients connect to them in order to gather information [60]. For the purpose of SEFU/IFU system design, an intelligent sensor implements an OPC UA server that comprises a self-description. This self-description includes device specific information such as the physical phenomena an intelligent sensor is capable of observing, a UID, etc. The self-description is modelled by SensorML. Here, intelligent sensors and their components (solid sensors and algorithms) are modelled by experts’ knowledge. Information that is required for the orchestration procedure (cf. Section 3.3) is deposited in the self-description and is available for the middleware.

The concept of SensorML is of syntactic nature. Hence, reasoning about compositions or other tasks, which require information about specific relations, is not possible offhand. Nevertheless, SensorML documents can be semantically extended, for example, by using XML *Linking Language* (XLink), i.e., a language for referencing (creation of hyperlinks) in XML documents [29]. The linkage, for example, to an ontology, extends the expressiveness of SensorML and improves knowledge derivation. A complete introduction to SensorML is found in [61].

**Figure 3:**
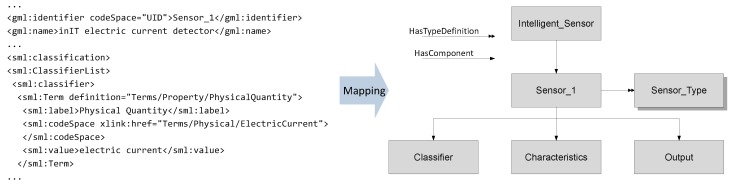
Excerpt of information specifying identifier, name, and observed physical quantity of an elementary sensor. The information is mapped from a SensorML description to OPC UA [52].

An OPC UA information model, which makes the self-description of intelligent sensors available for all system members, is implemented for SensorML classes. Therefore, a mapping between SensorML and OPC UA is carried out. An example is depicted in Figure 3. It shows an excerpt of the description of an elementary sensor (*Sensor_*1). The sensor is part of an intelligent sensor (*Intelligent_Sensor*) and has the definition type *Sensor_Type*. *Sensor_*1 can be described by further information such as a *Classifier* or *Characteristics* fields.

### 4.2. Fusion System Configuration and Adaptation

The design and configuration of adaptive SEFU/IFU systems requires different tasks to be performed. The sequence of those tasks is as follows [52]:

1. The system manager detects available intelligent sensors.
2. Semantic information is transferred to the KB.
3. Fusion system configuration is automatically carried out.
4. The system structure is observed to automatically adapt the fusion system.

**Figure 4:**
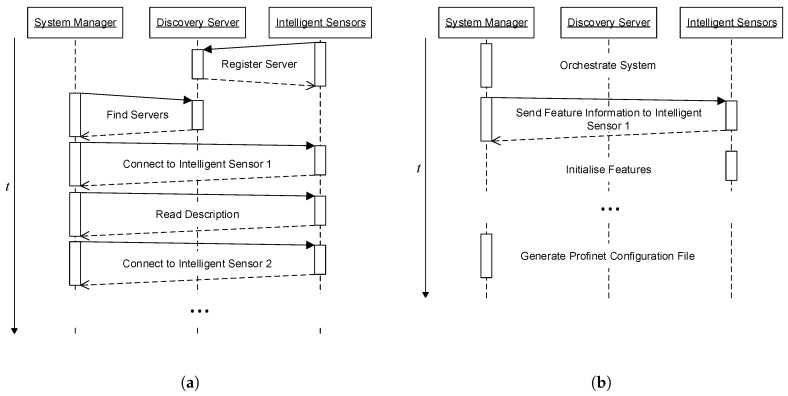
Communication sequences between system manager, discovery server, and intelligent sensors for SEFU/IFU system configuration. (**a**) Sequence describing the detection of intelligent sensors [52]; (**b**) Communication of feature information for assigning feature computation tasks to intelligent sensors.

In a first step, the system manager has to detect available intelligent sensors in the network. As no prior knowledge about present devices is available, a registration procedure is required. Therefore, OPC UA offers a *Local Discovery Server* (LDS), which provides the functionality to publicly expose available OPC UA servers in a local network. The LDS has to be available from one component of the SEFU/IFU network and its connection endpoints have to be available before carrying out the orchestration procedure. The sequence for intelligent sensor detection and exchange of semantic information is depicted in Figure 4a. Each intelligent sensor connects to the LDS after start-up and sends information for connection establishment. For detection of available intelligent sensors, the system manager queries the LDS. A connection is established to each device and its self-description is transferred to the knowledge base after the system manager is aware of available intelligent sensors. This procedure is repeated for each intelligent sensor.

Based on available information, a possible orchestration of the SEFU/IFU system is automatically inferred by the system manager. Sensors are assigned to algorithms resulting in features, attributes are generated depending on the set-up of the observed system and finally features are assigned to available attributes. While attribute fusion is applied by the system manager, the computation of features is carried out by the intelligent sensor that offers the corresponding algorithm. However, the associated sensor signal may be available from another intelligent sensor. Hence, the system manager sends that information to each intelligent sensor in order to initialise features. The sequence for feature initialisation is depicted in Figure 4b.

After the configuration of fusion system components (features and attributes) is completed, the real-time communication network of process data is established. This requires a configuration file depending on the actual fusion system orchestration. The configuration file is automatically generated by the system manager after sending feature initialisation data (cf. Figure 4b). The real-time communication of process data is detailed in the next section.

The fourth task aims to detect changes in the SEFU/IFU system (network). Intelligent sensors may be detached from the system for any reason. This causes an unavailability of specific measurements or algorithms that are required to guarantee proper information fusion. Hence, an adaptation of the fusion system with respect to the set of currently available intelligent sensors is required. Therefore, the LDS is periodically queried by the system manager. In case of a detachment (unavailability) of an intelligent sensor, its information is removed from the knowledge base and the orchestration procedure is executed again. The opposite case (the attachment of a new intelligent sensor) also triggers a re-orchestration of the SEFU/IFU system.

### 4.3. Process Data Communication

Depending on the specific application, process data is required to be communicated in real-time. Accordingly, intelligent sensors are equipped with a real-time communication interface (cf. Definition 5). Process data which is transmitted in real-time arrives deterministically at its destination within a pre-defined timeframe. In industrial environments, time-sensitive process data is generally transmitted using a *Real-Time Ethernet* (RTE) protocol. This contribution’s implementation relies on the *Profinet* standard for communication in real-time [62]. Its main characteristics are support of soft and hard real-time requirements and interoperability with standard Ethernet applications. Details about the utilised Profinet software stack are listed in Appendix A.

Communication is managed by a central entity in most RTEs. In Profinet, this entity is referred to as *I/O Controller*. The remaining network participants are called *I/O Devices*. For reasons of readability, the two are hereinafter referred to as controller and devices, respectively. In this implementation, the system manager hosts the controller, whereas the intelligent sensors function as devices. The main task of a controller is to collect and process all input data, e.g., sensor data, and to produce and transmit all output data, e.g., for actuators. This corresponds to a producer–consumer-model in which the controller is the producer of output data and the consumer of input data. Process data is communicated periodically. The controller defines the length as well as structure of communication frames and schedules the timing of all messages. A Profinet network needs at least one controller to be operable. In contrast to a controller, a device consumes output and produces input data. Devices are composed of modules. A module is a logical aggregation of process data, either input or output data. The configurations of all devices and their modules are defined in a *Generic Station Description* (GSD) file. It contains information for each device about how many input and output modules it has and about the data length of each module. This file is accessed by the controller to configure communication accordingly.

In the case of the proposed concept, intelligent sensors are required to send sensor signals, features and attributes to the system manager where the final fusion is carried out. Each single sensor signal, feature and attribute is assigned to one single module. Data which is to be sent to the controller or to other devices is modelled with an input module. Data which is sent from the controller and received by a device is represented by an output module. Furthermore, devices are required to send sensor signals and features to other specific devices for computation of additional features and attributes. As Profinet does not support a direct inter-device communication, this data is collected by the controller as well. The controller then forwards the data to the target device. For this, the configuration of input modules is extended by their required *destinations*. A destination describes the device at which the sensor signal or feature is needed for further computation. One input module can have multiple destinations.

**Figure 5:**
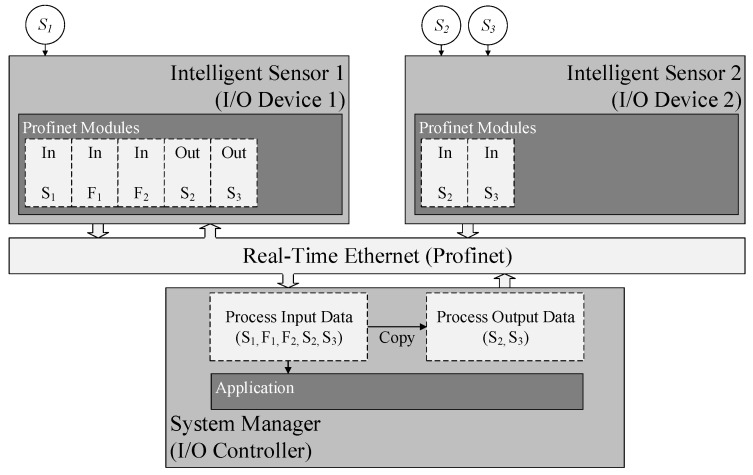
Profinet configuration of intelligent sensors in the fusion system network.

An exemplary Profinet network for a fusion system is depicted in Figure 5. The example consists of two intelligent sensors and the system manager. The first intelligent sensor (*device 1*) hosts one elementary sensor ($S_{1}$), the second (*device 2*) has access to two sensors ($S_{2}$ and $S_{3}$). For each elementary sensor, an input module is created. The first intelligent sensor offers algorithms to extract features $F_{1}$ and $F_{2}$ from signals $S_{2}$ and $S_{3}$. Therefore, these signals need to be transmitted from *device 2* to *device 1*. Because devices cannot communicate process data directly to each other, the signals of $S_{2}$ and $S_{3}$ are gathered by the system manager and are sent from there to *device 1*. For this, two input modules for the features and two output modules for the sensor signals are created at *device 1*. Furthermore, all gathered process input data is handed over to the fusion application by the system manager.

The devices of a Profinet network need to be configured dynamically for the specific orchestration. Different orchestrations require different sensor signals and features to be transmitted. Each intelligent sensor is modelled with an input module for every elementary sensor independent of the orchestration. The orchestration specifies (i) at which devices these sensor signals are additionally needed; (ii) which signals an intelligent sensor is required to receive; and (iii) which features it needs to send to the controller. Therefore, the Profinet configuration is carried out after the structure of the fusion system is determined. For this, the controller and every device go through a series of steps each.

The first step for the controller is to read the GSD file provided by the system manager. The controller then opens a non-real-time communication channel and waits for devices to connect. Configuration data is sent to devices via this channel. Simultaneously, the controller starts the Profinet service. The first step for devices is to connect to the non-real-time communication channel and request configuration data. After a device received its configuration, it accordingly creates modules, starts its Profinet service, and begins to communicate the process data in real-time.

As the structure of each device depends on the overall fusion system, it needs to be dynamically updated as soon as the fusion system is re-orchestrated. New or missing intelligent sensors are detected by the discovery server of OPC UA (cf. Section 4.2). As soon as the Profinet controller detects that the system manager updated the GSD file, it transmits the new configuration to each connected device. Afterwards, the controller as well as all devices restart their Profinet service.

## 5. Evaluation

The presented approach to automated fusion system design and adaptation is evaluated with respect to a printing unit demonstrator. The application aims to apply machine conditioning under laboratory conditions. A description of the application is given in the following example [6,11].

**Example 3** (Printing unit demonstrator)**.** Intaglio is the major printing process to produce security prints such as banknotes. Engraved structures in the printing plates, which are mounted on a rotating plate cylinder, are filled with ink, which is transferred onto the printing substrate under high pressure. A second cylinder, denoted as wiping cylinder, which is working in the printing unit, is lubricated with a solvent to wipe off surplus ink from the printing plates by rotating in the direction opposite to the plate cylinder.

**Figure 6:**
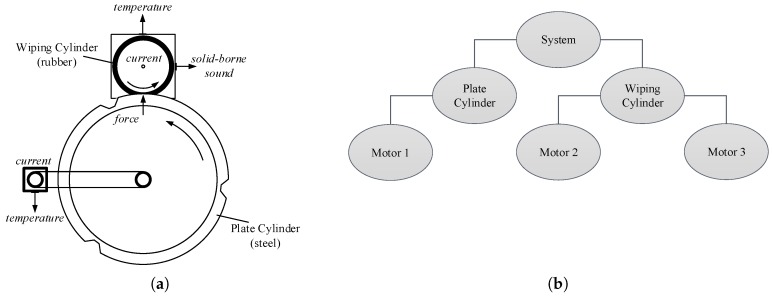
Set-up of the printing unit demonstrator. (**a**) Structural design of the printing unit demonstrator [6]; (**b**) Hierarchy of the demonstrator [7].

The printing unit demonstrator simulates the wiping process that consists of two cylinders. The plate cylinder is equipped with a single servomotor for rotation. The wiping cylinder is also driven by a servomotor, but consists of an additional linear drive for vertical positioning. This allows to freely adjust the pressure between the wiping cylinder and the plate cylinder. To monitor the process, seven analogue sensors (force, solid-borne sound, temperature, acoustic, electric current of each drive) observe the demonstrator. Its set-up is sketched in Figure 6a.

The hierarchy of the demonstrator is depicted in Figure 6b. The top-level represents the overall system. The sub-components are the wiping cylinder and the plate cylinder. The plate cylinder has got only one child node. This node is its drive, which is denoted by *motor 1*. As mentioned before, the wiping cylinder consists of a servomotor and a linear drive. These are denoted by *motor 2* and *motor 3*. The hierarchy of the overall system is necessary for an accurate orchestration. If, for example, a sensor observes *motor 1*, this information is also related to the plate cylinder and has to be considered for attribute assignments.

The applied solid sensors state different physical phenomena and are attached to particular objects of the demonstrator. Incorporated sensors are listed in Table 2. The table includes information about the physical phenomenon that the sensor states, the dimensionality of the output signal, and the sensor’s associated object.

For feature computation, algorithms are available from the intelligent sensors. For the evaluation use case scenario, two algorithms are incorporated. These are detailed in Table 3. Algorithm $A_{1}$ is the mean operator, whereas algorithm $A_{2}$ is the variance operator. Restrictions have to be considered for both algorithms. On the one hand, they are only applicable to specific physical phenomena. On the other hand, input signals have to be of a certain dimensionality.

The printing unit demonstrator is equipped with three intelligent sensors. Table 4 shows the set of available intelligent sensors and the implemented solid sensors and algorithms.

### 5.1. Orchestration

The printing unit demonstrator and its equipment of intelligent sensors is used for evaluation. First, features are inferred by applying the forward chaining algorithm with respect to the rules defined in Section 3.3.2. Sensors $S_{j}$ are assigned to algorithms $A_{k}$ resulting in a set of features F. Table 5 lists the set of inferred features with respect to the previously mentioned system set-up.

The outputs of the features represent the inputs for subsequent fusion operators. For the MACRO fusion system, features are assigned to meaningful attributes. In Section 3.3.1, a procedure for the initialisation of possible attributes is introduced. It relies on the system hierarchy and the set of available solid sensors. The initialisation is carried out before features are assigned to some attributes. Rules for the assignment of features to attributes are defined in Section 3.3.3. Applying the forward chaining algorithm to these rules results in a set of associated features $C_{a_{i}}$ for each attribute $a_{i}$. After the algorithm terminates, elements with $|C_{a_{i}}|\leq 1$ are rejected from $C_{a_{i}}$. This is required to obtain only meaningful attributes as the fusion of only single inputs is not possible. Finally, remaining attributes $a_{i}$ show a possible design for the fusion system and are proposed to the system designer. The result for the set-up of the printing unit demonstrator is depicted in Table 6. Two physical attributes $a_{1}$ and $a_{2}$ are inferred, whereas attributes $a_{3}$ and $a_{4}$ are module attributes that are related to the wiping and the plate cylinder. Attribute $a_{5}$ is a manually defined functional attribute. This is carried out by the system designer, who is an expert of the observed system. In this case, the *running smoothness* of the system is modelled as a functional attribute. Allowed physical phenomena for this attribute are acoustic, current consumption, and solid-borne sound. The mean value as well as the variance value of sensor measurements are allowed for this attribute (cf. Example 2). Consequently, four features are assigned to it (cf. Table 6).

### 5.2. Fusion System Update

**Figure 7:**
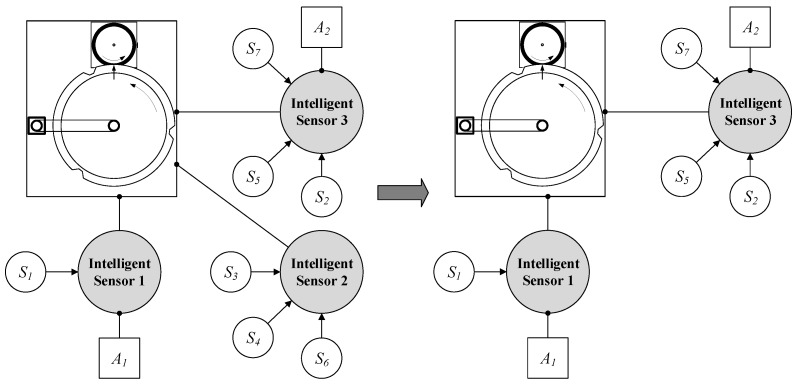
Rejection of one intelligent sensor from the fusion system architecture.

The orchestration results in a system design that is related to the actual set-up of available intelligent sensors. Because of the trend towards distributed and modular systems, the set-up may change. This has to be considered for the design of the SEFU/IFU system. The fusion system has to be adapted to the actual system structure either periodically or at defined times. Consider the previously mentioned scenario. At time $t_{0}$, three intelligent sensors that observe the printing unit demonstrator are available. At a later time $t_{1}$, one intelligent sensor shows a defect or is detached from the system. Hence, the intelligent sensor is not available for the fusion system anymore. This is depicted in Figure 7. IntelligentSensor2 is removed from the fusion system. Therefore, its associated solid sensors ($S_{3}$, $S_{4}$, $S_{6}$) can no longer provide its measurements to the fusion process.

The detection of changes is handled by the implemented middleware (cf. Section 4.2). It queries the LDS of OPC UA that detects changes in the system structure and adapts its list of available devices automatically. Therefore, the system manager is aware of the set of available intelligent sensors. After a change is detected, the orchestration system needs to update the attributes of the MACRO system. Consider the example of Figure 7. Sensors and features, which are not available, have to be removed and should not be considered for information fusion. Furthermore, attributes may become meaningless after the system structure has changed because of unavailable input data. Consequently, a re-orchestration is carried out after the system has changed. In this example, three solid sensors are not available anymore. Hence, the set of available features changes. Table 7 shows the new set of features F that is inferred by a re-orchestration.

The availability of attributes $a_{i}$ changes also with respect to the decremented set of features. For the use case of Figure 7, only two attributes are available after the system update. Attributes and their associated features are listed in Table 8.

Besides the removal of components, newly plugged intelligent sensors are detected, registered, and incorporated in the fusion system automatically. This case is different, since new information sources may enable to compute additional features or generate further attributes for the overall fusion system. The attachment of additional sources causes a re-execution of the orchestration procedure.

Subsequent to the derivation of features and attributes, the underlying communication of process data has to be reconfigured, too. Therefore, a new configuration file for the Profinet application is generated by the system manager. It includes information about data access points for sensor signals as well as feature and attribute inputs and outputs. The time-stamp of the configuration file is observed in order to automatically execute a reconfiguration of real-time communication channels. Hence, the entire SEFU/IFU system automatically adapts after a system update and gets into working state without any external configuration.

### 5.3. Discussion

Consider the aforementioned scenario of the printing unit demonstrator. Even if the overall system is not that complex, it is still time-consuming for humans to understand and model the complete system structure and to identify relations of available components. Furthermore, the fusion system designer has to be aware of the monitored system and available modules. By the application of self-descriptive intelligent sensors, the approach to automated fusion system design and adaptation relieves the user of the task. Sets of possible features and attributes are inferred and proposed to the user. This provides the user an overview of the monitored system, even if there is no prior knowledge about it. The assignment of features to attributes identifies relations between them and results in a possible fusion system design. The identification of relations is a highly complex and time-consuming task with respect to the number of features and attributes. Following the presented approach, a possible fusion system is proposed, which again improves the comprehensibility of the system with respect to condition monitoring. Therefore, less qualified users are also able to set up and maintain fusion systems.

Rule-based systems are powerful for knowledge modelling and inference. In combination with SensorML, an expressive knowledge base is generated. Nonetheless, the prototypical implementation of the developed orchestration system relies on term definitions that ensure a common semantic. Hence, the system is currently restricted to those terms. If a description file includes an unknown term, no inference can be drawn. Therefore, the system is limited with respect to the description of semantic relations.

Related approaches from the area of sensor orchestration put their focus in other directions. They concentrate on tasks such as sensor or service discovery or composition, but do not consider aspects of fusion systems. Furthermore, the concept of intelligent sensors, which will be available on the future market, is not considered by related works. Only solid sensors or sensor nodes are incorporated in existing approaches. Even if sensor nodes include processing units and consist of a set of solid sensors, they are different to the concept of intelligent sensors. Sensor nodes do not include a self-description and their processing units mainly focus on data exchange and networking.

Iswandy and König have developed a complete design methodology for multi-sensor systems [3]. Their system includes concepts for sensor and feature selection but relies on a different mathematical background. They utilise *genetic algorithms* and *particle swarm* optimisation. Their approach focuses on sensor signals for the sensor selection process and does not include any description of sensors. Therefore, semantic relations among these are not considered by their approach. With respect to intelligent sensors, which implement local intelligence and are capable of self-description, the sequential optimisation procedure is not suitable. In addition, their methodology completely replaces humans in the design process. As mentioned before, humans should serve as final decision makers and include their implicit and explicit knowledge into the SEFU/IFU system design process. This aspect is not considered by the authors.

The approach proposed by Compton et al. is related to the task of automated sensor orchestration, but does not focus on the use case of information fusion systems [30]. Their approach is capable of sensor-classification and identification of possible compositions of sensors in order to generate high-level information. They have developed an OWL ontology for sensor specific information and apply reasoning inside OWL. Although their ontology is expressive, sensors cannot be modelled precisely in OWL and require external definitions realised by concepts of *Description Logic* (DL) [30]. The presented concept of this work relies on SensorML and on simple but effective logic rules for knowledge inference. Because of the extensible structure of SensorML and the precise formulation of knowledge by rules, the orchestration concept offers similar capabilities but is more lightweight than the approach of Compton et al. Furthermore, their concept considers only compositions in terms of feature generation, but does not investigate the orchestration of sensors into reasonable groups.

Another related approach is presented by Loskyll et al. [36]. Instead of sensor orchestration, the authors focus on the orchestration of services of automation systems. Therefore, they specify an ontology and extend service descriptions by semantic annotations using the *Semantic Annotation for Webservices Description Language* (SAWSDL) and OWL. SAWSDL is a description language that arises from the area of SWSs where it is used for referencing to semantic models. Their system supports the user in the orchestration of services by semantic matchmaking, i.e., it suggests suitable services. Hence, the user is guided during the orchestration process. Their matching procedure could also be applied to the task of sensor orchestration, but it contains drawbacks compared to the orchestration concept elaborated here. Similar to [30], the system relies on a complex ontology. An orchestration system does not have to include complex knowledge representations. The required functionalities can even be fulfilled by more effective and lightweight concepts. As mentioned before, ontology engineering is a time-consuming process that requires high-skilled operators. Therefore, a rule-based system is easier to handle and maintain. Furthermore, as the formulation of rules is simple for human operators, the system is extensible offhand.

Another concept is proposed in [34]. Its field of work is sensor networks. The aim is to assign specific roles to sensors in order to improve aspects such as energy consumption, life-time, or coverage. Instead of an accurate description of sensors, the assignment relies on user-defined roles that are available at sensor nodes. The latter is comparable to intelligent sensors, i.e., sensor nodes containing a communication module and processing unit. A role assignment algorithm is available at every sensor node and used to infer the particular role of this node. The algorithm relies on formulations of predicate logic and evaluates rules for role assignment. Although this is similar to the orchestration procedure of this contribution, their concept is different. The authors specify roles externally and propagate these to available sensor nodes that implement the logic for role assignment. Here, intelligent sensors are incorporated for orchestration and available knowledge is collected in a shared knowledge base. With respect to information fusion systems, this is a superior concept since knowledge about the overall system is available from a central repository. SEFU/IFU systems consist of a connection of heterogeneous information sources. These are easier to handle and overview in a central knowledge base instead of a distributed network. Hence, a central knowledge base simplifies fusion system generation and maintenance.

Besides the functionality of the orchestration concept and its advantages compared to related approaches, its complexity is of interest. The orchestration engine relies on a rule-based system that infers knowledge from given rules and facts using the forward chaining algorithm (cf. Section 3.2). Rules defined for orchestration are independent of each other, i.e, inferred facts do not affect other rules. Thus, the forward chaining algorithm is required to iterate over the rule base only once. The computational complexity of the orchestration is consequently $O\left(N\right)$ for N→∞, in which *N* is the number of rules in the knowledge base. The computational steps necessary for the orchestration grow linearly in *N*.

The number of rules depends on the number of available elementary sensors and algorithms. Rules are added to the rule base R for the assignment of features and attributes. For feature assignment, the rule base contains rules for all possible combinations of algorithms A and elementary sensors S. Thus, the amount of rules added to R for the assignment of features is

$$|R_{F}|=|S|\cdot |A|.$$

Furthermore, R contains rules for the assignment of attributes. They are added for each possible combination of features F and attributes A. Hence, the amount of rules added for the assignment of attributes is

(4) $$|R_{A}|=|F|\cdot |A|.$$

Computational complexities are evaluated in a worst case scenario. The complexity states that an algorithm does not perform worse than a comparison function. Here, a scenario is considered as worst case if a maximum of rules is generated in an orchestration. The number of rules in $R_{A}$ is maximal if an orchestration identifies a maximum number of features and attributes.

Features are created by applying an extraction algorithm to a sensor signal. As detailed in Section 3.3.2, the input specifications of an algorithm and a sensor are required to match. In the worst case, all sensors match to all available algorithms. Thus, the maximum amount of features is equal to the amount of elementary sensors times the amount of available algorithms:

(5) $${\left|F\right|}_{\max}=\left|S\right|\cdot |A|.$$

The maximum amount of attributes relies on the maximum number of features. In the following, only module and physical attributes are considered. Since functional attributes are designed manually, their number cannot be estimated. Quality attributes are treated by the orchestration as a special case of a module attribute. A sensor monitors either a module or the fabricated product. With regard to Table 5, a sensor contributing to a quality attribute has the associated object *product*. Each feature monitors only one single module or the product. It also captures only one single physical phenomenon. Consequently, it can only be part of two attributes, either a module or quality attribute and one single physical attribute. Considering that at least two features are required to form an attribute as specified in Definition 3, an orchestration results in the maximum amount of attributes if all features pair up once for module and once for physical attributes. The maximum amount of attributes, again without considering functional attributes, is then formalised as follows:

(6) $${\left|A\right|}_{\max}=\left\lfloor \frac{{\left|F\right|}_{\max}}{2}\right\rfloor \cdot 2.$$

Considering Equations (4)–(6), the maximum amount of attribute rules is

$$\begin{array}{cc} |R_{A}{|}_{\max} & =\left|S\right|\cdot \left|A\right|\cdot \left\lfloor \frac{\left|S|\cdot |A\right|}{2}\right\rfloor \cdot 2\\ & \leq {\left(|S|\cdot |A|\right)}^{2}. \end{array}$$

The maximum total amount of rules in the rule base is the summation of $|R_{F}|$ and $|R_{A}{|}_{\max}$:

$$\begin{array}{cc} {\left|N\right|}_{\max} & =|R_{F}\left|+\right|R_{A}{|}_{\max}\\ & \leq \left|S\right|\cdot \left|A\right|+{\left(|S|\cdot |A|\right)}^{2} \end{array}$$

The growth of the number of rules is therefore in the worst case quadratically in both number of sensors ($O(|S{|}^{2})$ for |S|→∞) and number of algorithms ($O(|A{|}^{2})$ for |A|→∞). The number of rules is independent of the amount of implemented intelligent sensors.

## 6. Conclusions and Outlook

This article proposes an approach to the automated design of adaptable SEFU/IFU systems. Intelligent sensors, i.e., self-descriptive modular devices that consist of one or more elementary sensors, a processing unit and communication interfaces, are the basic elements that form a fusion network for system monitoring. A system manager detects available intelligent sensors. Semantic data is exchanged by a middleware. An orchestration procedure that relies on a knowledge-based system in the form of a rule-based system is introduced. It automatically deduces features and attributes for the SEFU/IFU system with respect to the hierarchy of the monitored system and the set of available intelligent sensors. This results in a possible solution for the design of a SEFU/IFU system. The system designer remains the final decision maker in this process. Furthermore, the underlying real-time communication system for process data exchange is configured automatically with respect to the actual design of the fusion system. Both the orchestration and the configuration of communication channels relieve the system designer during design and set-up of the SEFU/IFU system. The middleware is capable of detecting changes in the system during run-time (insertion and rejection of intelligent sensors) and adapting the SEFU/IFU system automatically with respect to the actual set of available data sources. The presented approach for SEFU/IFU system design is evaluated with respect to a sample scenario in the scope of a printing unit demonstrator.

The current implementation of the proposed automated fusion system design relies on several centralised entities and repositories. First of all, the system manager represents a central unit. It is divided into an orchestration engine and a knowledge base. The knowledge base consists of a central rule base and various repositories (sensor, feature, attribute, and algorithm repositories). Furthermore, the communication of process data is centrally organised as well. It relies on a central Profinet controller, which manages the communication process.

Merging the proposed automated fusion system design with concepts of MASs and self-organising distributed systems is an open research topic for future work. In such an approach, the orchestration and the information fusion system themselves become decentralised. The aim is to increase the fault tolerance, scalability, and accessibility of the automated design system. A self-organising distributed fusion system without a central control unit requires autonomously acting sensor nodes. Intelligent sensors, as introduced in Definition 5, provide local processing and communication capabilities, are self-aware and provide self-configuration. Because of these capabilities, an intelligent sensor is well-suited to function as an embodied agent (cf. Section 2). Research on agents focuses on individual decision-making processes, i.e., how an agent decides to act in and react to an environment. MAS technologies focus on social abilities, self-organisation, cooperation, negotiation, and decision-making of a collective of individual agents. Therefore, extending the intelligent sensor with agent capabilities is a promising possibility to decentralise the orchestration and SEFU/IFU system. An agent-based intelligent sensor relies on a local knowledge base rather than on a globally available knowledge base. The local knowledge base is a symbolic representation of the intelligent sensor’s individual knowledge about its environment (i.e., which agent hosts which sensors, features, and algorithms).

Furthermore, a distributed self-organising orchestration system cannot rely on a central discovery server such as the LDS utilised in this work. Intelligent sensors (as agents) need to explore the fusion network themselves. For this, it is crucial that an agent searches for, discovers and evaluates potential partners efficiently [63]. Partners are required for the orchestration tasks of attribute initialisation, feature inference and feature assignment. In the final fusion system, cooperation is also required for the computation of features and attributes.

In order to be able to evaluate potential partners, a concept for the exchange of information needs to be elaborated. Possible approaches are peer-to-peer requests or broadcasting requests [63]. Peer-to-peer requests promise a lower communication load and are more flexible to use. Broadcasting is more easy to implement, but non-trivial and expensive especially in wireless ad-hoc networks. A peer-to-peer approach, on the other hand, introduces the possibility that an intelligent sensor does not find the information it is looking for. This results in a possibility that the orchestration does not identify all possible features or attributes.

The collective of intelligent sensors (the MAS) is required to find an optimal organisation. The organisation of a distributed system is the sum of allocated tasks, relationships between nodes and authority structures [64]. The organisation of a system immediately influences its performance. Unsuitable designs unnecessarily increase computational and communication complexity of a system, and reduce flexibility and reactivity at the same time.

Furthermore, every decision has to be made collectively in a self-organised distributed system. Existing approaches to collective decision-making are based on voting models [43], biologically inspired swarm methods [65,66,67], or task and role allocation methods [68].

A major challenge for decentralised information fusion systems is real-time communication. State-of-the-art RTE protocols, such as Profinet, require a central control unit which manages the timing and flow of communication. This challenge needs to be considered and met in future approaches to a decentralised information fusion system.

mystyle

## Figures and Tables

**Table 1:** Heterogeneity of acquired data in a Sensor and Information Fusion application in terms of data characteristics.

*Characteristic*	*Occurrence*	
quantity	physical (pressure, temperature, speed, etc.)	non-physical (expert knowledge, manufacturing inventory, production rate, etc.)
value domain	continuous	discrete
codomain	R	binary, multi-valued
time domain	continuous	discrete
sampling	—	equidistant, non-equidistant (including event-triggered)

**Table 2:** Available sensors and their characteristics.

Solid Sensor	Physical Phenomenon	Dimensionality	Associated Object
$S_{1}$	temperature	1	Motor 1
$S_{2}$	temperature	1	Motor 2
$S_{3}$	current consumption	1	Motor 1
$S_{4}$	current consumption	1	Motor 2
$S_{5}$	solid-borne sound	1	Wiping Cylinder
$S_{6}$	contact force	1	Wiping Cylinder
$S_{7}$	acoustic	1	System

**Table 3:** Available algorithms and their characteristics.

Algorithm	Type	Physical Phenomena	Input Dimensionality
$A_{1}$	mean operator	temperature, current consumption	1
$A_{2}$	variance operator	acoustic, solid-borne sound, contact force	1

**Table 4:** Intelligent sensors and their equipment.

Intelligent Sensor	Solid Sensors	Algorithms
Intelligent Sensor 1	$S_{1}$	$A_{1}$
Intelligent Sensor 2	$S_{3},S_{4},S_{6}$	∅
Intelligent Sensor 3	$S_{2},S_{5},S_{7}$	$A_{2}$

**Table 5:** Generated features of the orchestration procedure.

Feature	Sensor	Algorithm	Algorithm Type	Physical Phenomenon	Associated Object
$F_{1}$	$S_{1}$	$A_{1}$	mean operator	temperature	Motor 1
$F_{2}$	$S_{2}$	$A_{1}$	mean operator	temperature	Motor 2
$F_{3}$	$S_{3}$	$A_{1}$	mean operator	current consumption	Motor 1
$F_{4}$	$S_{4}$	$A_{1}$	mean operator	current consumption	Motor 2
$F_{5}$	$S_{5}$	$A_{2}$	variance operator	solid-borne sound	Wiping Cylinder
$F_{6}$	$S_{6}$	$A_{2}$	variance operator	contact force	Wiping Cylinder
$F_{7}$	$S_{7}$	$A_{2}$	variance operator	acoustic	System

**Table 6:** Resulting attributes of the orchestration.

Attribute	Attribute Type	Characteristic	Associated Object	$C_{a_{i}}$
$a_{1}$	physical	temperature	System	$\left\{F_{1},F_{2}\right\}$
$a_{2}$	physical	current consumption	System	$\left\{F_{3},F_{4}\right\}$
$a_{3}$	module		Wiping Cylinder	$\left\{F_{2},F_{4},F_{5},F_{6}\right\}$
$a_{4}$	module		Plate Cylinder	$\left\{F_{1},F_{3}\right\}$
$a_{5}$	functional	running smoothness	System	$\left\{F_{3},F_{4},F_{5},F_{7}\right\}$

**Table 7:** Generated features of the orchestration procedure.

Feature	Sensor	Algorithm	Associated Object
$F_{1}$	$S_{1}$	$A_{1}$	Motor 1
$F_{2}$	$S_{2}$	$A_{1}$	Motor 2
$F_{3}$	$S_{5}$	$A_{2}$	Wiping Cylinder
$F_{4}$	$S_{7}$	$A_{2}$	System

**Table 8:** Resulting attributes after system update.

Attribute	Attribute Type	Characteristic	Associated Object	$C_{a_{i}}$
$a_{1}$	physical	temperature	System	$\left\{F_{1},F_{2}\right\}$
$a_{2}$	functional	running smoothness	System	$\left\{F_{3},F_{4}\right\}$

## Abbreviations

The following abbreviations are used in this article:

AutomationML: Automation Markup Language
DL: Description Logic
GSD: Generic Station Description
KB: Knowledge Base
LDS: Local Discovery Server
MACRO: Multilayer Attribute-based Conflict-reducing Observation System
MAS: Multi-Agent System
OPC UA: Open Platform Communication Unified Architecture
OWL: Web Ontology Language
RTE: Real-Time Ethernet
SAWSDL: Semantic Annotation for Webservices Description Language
SEFU/IFU: Sensor and Information Fusion
SensorML: Sensor Model Language
SWS: Semantic Web Services
UID: Unique Identifier
XLink: XML Linking Language
XML: eXtensible Markup Language

## Appendix A. Implementation Specifications

### Appendix A.1. Raspberry Pi

The incorporated device is a Raspberry Pi Model 3B. The operating system is Raspbian Jessie with release date 2016-05-27, based on Debian Jessie [57].

### Appendix A.2. OPC UA

A free Java OPC UA software development kit, certified by the OPC Foundation to be OPC UA 1.02 compliant, is available from Prosys [69]. The implementation relies on version 2.1.0-436.

For information modelling, the *UaModeler* software available from the *Unified Automation GmbH* is incorporated in version 1.4.1 [70].

### Appendix A.3. SensorML

A free Java application programming interface and implementation of a SensorML-based processing engine is incorporated [71].

### Appendix A.4. Profinet Controller/Device Stack

A proprietary software for the implementation of Profinet controllers and devices developed by Phoenix Contact is applied in the implementation of this article. The utilised version 3.8.33.7 is specifically designed for Linux operating systems.
